# Early onset neutropenia: a useful predictor of chemosensitivity and favorable prognosis in patients with serous ovarian cancer

**DOI:** 10.1186/s12885-020-6609-x

**Published:** 2020-02-12

**Authors:** Yijing He, Ting Li, Jue Liu, Qiong Ou, Junlin Zhou

**Affiliations:** 1 Department of Obstetrics and Gynecology, The Second Affiliated Hospital of University of South China, Hengyang, China; 2 Clinical Research Institute, The First Affiliated Hospital of University of South China, Hengyang, China

**Keywords:** Timing of onset of chemotherapy-induced neutropenia (CIN), Chemotherapeutic response, Prognosis, Serous ovarian cancer

## Abstract

**Background:**

Epithelial ovarian cancer (EOC) is the leading cause of gynecological cancer-associated deaths and a majority of its histological type is manifested as serous ovarian cancer (SOC). In this study, we investigated whether the timing of onset of chemotherapy-induced neutropenia (CIN) is related to chemotherapeutic response and disease outcome of SOC.

**Methods:**

One hundred sixty-nine primary SOC patients receiving six doses of carboplatin plus paclitaxel adjuvant chemotherapy following cytoreductive surgery were retrospectively included in this research. CIN was grouped as early onset and late onset neutropenia depending on the timing of development. Development of CIN prior to or with administration of 3rd cycle of chemotherapy was listed as early onset neutropenia, while those CIN due to later stage chemotherapy were grouped into non-early type. The relevance of time of CIN onset with the clinical characteristics, chemotherapeutic response, progression free survival (PFS) and overall survival (OS) were determined and analyzed by using Kaplan–Meier curves, Logistic regression method, Cox proportional hazards models, and Chi-square tests.

**Results:**

The age distribution of the patients was between 27 to 77 years. Fifty years was the median. No statistical significances of difference in age, FIGO stage, histological grade, tumor residual and lymph node invasion, as well as CA125 level in each CIN group were found (all *P*>0.05). The patients from non-early onset group showed higher chemoresistance rates (78.33%) compared to those from early onset group (9.17%). Additionally, patients in early onset group showed improved median PFS (23 vs. 9 months; *P*<0.001) and median OS (55 vs.24 months; *P*<0.001).

**Conclusions:**

Early onset neutropenia may be potentially used as a potential indicator for chemosensitivity and favorable prognosis of SOC in patients who underwent six cycles of carboplatin plus paclitaxel adjuvant chemotherapy following primary cytoreductive surgery.

## Background

Epithelial ovarian cancer (EOC) is the leading cause of gynecological cancer-associated deaths and a majority of its histological type is manifested as SOC [1]. Despite high clinical response rate, recurrences of the SOC post primary combined surgery and chemotherapy are common. Majority of the relapses accompany non-responsiveness to further chemotherapy which eventually leads to death [2, 3]. Although, in recent years, some studies have attempted to reveal the prognostic factors and biomarkers for prediction of responses to chemotherapy and survival, the application of such prediction parameters are still limited. Therefore, identification of an easy and reliable prognostic biomarker for disease surveillance and stratification of ovarian cancer is essential.

Neutropenia is a frequent adverse reaction following chemotherapy. The risk of developing neutropenia following the standard chemotherapy for EOC with carboplatin and paclitaxel is approximately 30–90% [4]. Despite being an adverse effect of chemotherapy, several researches have reported that CIN can be used for prediction of a favorable prognosis in different carcinomas of the breast [5], non-small cell lung [6], gastric [7], pancreas [8], and colorectum carcinoma [9, 10]. The association between CIN and the progress of ovarian cancer has been controversial. While, Kim et al. [11] suggested CIN as non-significant prognostic indicator in ovarian cancer, studies by Tewari et al. [4], the indicate improved survival rate in patients with CIN as compared to those patients who do not develop CIN. Recently, several studies reported that timing of CIN may predict chemotherapeutic response or survival [12–16]. However, the role of timing of CIN onset for predicting chemotherapeutic response and clinical outcome has not been evaluated for SOC patients.

Therefore, this research aims to determine the correlation of CIN onset and the response to chemotherapy, with carboplatin and paclitaxel, in terms of chemosensitivity and survival.

## Methods

### Patients and data collection

This retrospective study comprises of patients diagnosed with SOC and were admitted in the Second Affiliated Hospital of University of South China during the period between January, 2011 to June, 2013. The approval for the research was provided by the ethical committee of Second Affiliated Hospital of University of South China. Before study, written informed consents were obtained from the patients. All treatments and blood tests were performed according to institutional guidelines. The clinical records were gathered from the database of Second Affiliated Hospital of University of South China.

The criteria for inclusion in the study were as follows:1) histological or cytological confirmation of developing SOC and without prior treatment, such as radiotherapy or chemotherapy; 2) patients underwent cytoreductive surgery followed by carboplatin plus paclitaxel adjuvant chemotherapy; 3) normal bone marrow profile; 4) normally functioning liver and kidney. The exclusion criteria were: 1) incomplete record of toxicities; 2) lost follow-up; 3) second malignancies or multiple primary malignancies; 4) primary treatment in other hospital. One hundred sixty-nine SOC patients were found fit as per the criteria set for inclusion and exclusion in the present research.

### Dose intensity of chemotherapy

Chemotherapy regimens for all the patients were initiated within 4 weeks after primary cytoreduction. Each dose of carboplatin and paclitaxel comprise of (AUC = 5) and (175 mg/m^2^) respectively and were administered intravenously six times with a gap of 3 weeks.

### Assessment of neutropenia

Blood samples were collected both before (day 0 or day 1) and on every 7 days after initiation of chemotherapy. The development of CIN of the highest grade was used for analysis. CIN grading were carried out according to ruling of the National Cancer Institute (NCI) Common Terminology Criteria for Adverse Events (CTCAE, version 4.0). Grade 1, 2, 3 and 4 were assigned based on absolute neutrophil count (ANC) limit of 1.5 × 10^9^/L to 2.0 × 10^9^/L; 1.0 × 10^9^/L to 1.5 × 10^9^/L; 0.5 × 10^9^/L to 1.0 × 10^9^/L; less than 0.5 × 10^9^/L respectively. Grade 1 and 2 represent mild neutropenia, while grade 3 and 4 are denoted to severe form of neutropenia. Moreover, depending on minimum number of chemotherapeutic dose for development of CIN, they were listed as early onset and late onset neutropenia. Early onset group experience ANC fall less than2.0 × 10^9^/L with chemotherapy cycle 1–3, while in non-early onset group ANC level did not fall below2.0 × 10^9^/L until 4th cycle of chemotherapy. The use of granulocyte colony-stimulating factor (G-CSF) for prophylaxis was prohibited unless ANC reached below 0.5 × 10^9^/L.

### Follow-up

All patients enrolled in this study were regularly followed-up every 3 months until June 30, 2018 to obtain recurrence and survival information. Follow-up included a complete history of the disease, physical examination, blood tests, abdominal ultrasonography, CT scan of the chest and abdomen to exclude recurrence and metastasis. Recurrence was evaluated as per the guidelines of response evaluation criteria in solid tumors (RECIST) [17]. Development of progressive disease before 6 months of initial treatment were grouped as chemoresistant; while the others were grouped as chemosensitive [18]. PFS is determined by the time from the surgery to disease progression, while OS represents the time duration between cytoreductive surgery and death or, as the case may be, date of latest follow-up.

### Statistical analysis

Statistical differences between groups were determined using Wilcoxon and Pearson’s Chi-Square tests. Logistic regression method was applied for prediction of independent risk factors of chemoresistance. Survival curves were analyzed by the Kaplan–Meier curves and the log-rank test. Analysis of multivariates were done by Cox proportional hazards regression models. If the value *P* was found to be less than 0.05, then difference in the groups were considered statistically significant. The SPSS, version 23.0 (Chicago, IL, USA) software tool was used for all the statistical analysis.

## Results

### Patient demographics

A total of 169 patients with histologically identified SOC, who underwent cytoreductive surgery followed by carboplatin plus paclitaxel adjuvant chemotherapy, were eligible for this analysis. Table 1 showed clinical variables and the timing of CIN of the 169 patients. The median age of the patients was 50 years (range 25–77 years). Among 169 patients, 109 (64.50%) experienced early onset and 60 (35.50%) experienced non-early onset neutropenia. One hundred fifteen developed mild and the remaining 38 developed severe neutropenia. There were no significant differences in age, FIGO stage, histological grade, tumor residual and lymph node invasion, as well as CA125 level among groups by timing of CIN (all *P*>0.05) (Table 1).

**Table 1:** Clinical characteristics of patients by timing of CIN in patients with serous ovarian cancer(*n* = 169)

Variables	n	Early onset	Non-early onset	*P* value
Age (year)				0.839
<50	75	49	26	
≥ 50	94	60	34	
FIGO stage				0.462
I-II	45	27	18	
III-IV	124	82	42	
Histological grade				0.128
Low	42	23	19	
High	127	86	41	
Lymph node invasion				0.133
Negative	132	89	43	
Positive	37	20	17	
Tumor residual (cm)				0.058
Optimal(≤1)	127	87	40	
Sub-optimal(>1)	42	22	20	
CA125 level (U/mL)				0.634
≤ 35	12	9	3	
>35	157	100	57	
Severity of CIN				<0.001
Absence	16	0	16	
Mild	115	83	32	
Sever	38	26	12	

### The timing of CIN and chemoresistance

Table 2 showed clinical characteristics predicting chemoresistance. In this study, 57 out of 169 patients (33.73%) were found to be chemoresistant. The patients in non-early onset group have higher chemoresistance rates (78.33%) compared to the early onset group (9.17%). Besides, histological grade, severity of CIN was associated with chemotherapeutic response.

**Table 2:** Association between chemotherapeutic response and clinical characteristics

Variables	Chemotherapeutic response		*P* value
	Chemosensitive(*n* = 112)	Chemoresistance(*n* = 57)	
Age (year)			0.923
<50	50	25	
≥ 50	62	32	
FIGO stage			0.665
I-II	31	14	
III-IV	81	43	
Histological grade			0.001
Low	19	23	
High	93	34	
Lymph node invasion			0.550
Negative	89	43	
Positive	23	14	
Tumor residual (cm)			0.286
Optimal(≤1)	87	40	
Sub-optimal(>1)	25	17	
CA125 level (U/mL)			0.729
≤ 35	9	3	
>35	103	54	
Severity of CIN			<0.001
Absence	1	15	
Mild	81	34	
Sever	30	8	
Timing of CIN			<0.001
Early onset	99	10	
Non-early onset	13	47	

Furthermore, use Logistic analysis to assess the predictive significance of timing of CIN (Table 3) revealed that the non-early onset CIN was an independent predictor of chemoresistance [odds ratio (OR) 36.371, 95% confidence interval (CI) 12.364–106.993; *P*<0.001].

**Table 3:** Logistic analysis of the association between chemoresistance and timing of CIN

Variable	OR	95% CI	*P* value
Histological grade (high vs low)	0.188	0.062–0.567	0.003
Timing of CIN (Non-early vs Early)	36.371	12.364–106.993	<0.001
Severity of CIN			0.074
Sever vs Absence	0.085	0.009–0.852	0.036
Mild vs Absence	0.223	0.025–2.010	0.181
Mild vs Sever	2.606	0.784–8.667	0.118

### Survival analysis

**Fig. 1:**
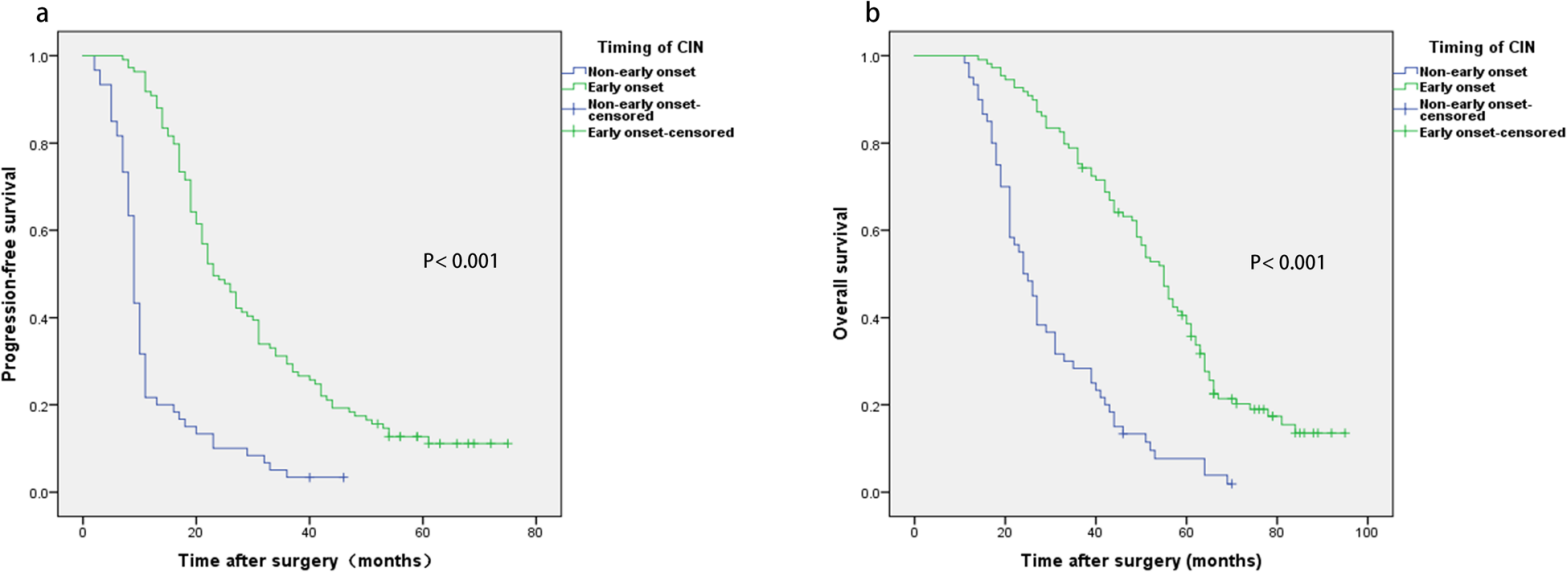
Kaplan–Meier survival *curves* demonstrating relationships between timing of CIN and PFS (**a**) and OS (**b**) of patients with SOC

The patients had a median PFS of 19 months and a median OS of 44 months. There was a significant association between timing of CIN and survival using Kaplan−Meier analysis. As shown in Fig. 1, the early onset group showed significantly higher PFS and OS than the non-early onset group. The median PFS in early and non-early onset groups were 23 and 9 months, respectively (*P*<0.001), while the median OS were 55 and 24 months, respectively (*P*<0.001).

To assess the prognostic significance of timing of CIN, we performed the univariate and multivariate Cox regression analysis. According to Table 4, univariate analysis focused on several variables of survival, including age, FIGO stage, histological grade, tumor residual, lymph node invasion, CA125 level, severity of CIN, and the timing of CIN. FIGO stage (*P*<0.001), tumor residual (*P* = 0.003), lymph node invasion (*P* = 0.013), CA125 level (*P*<0.001), timing of CIN (*P*<0.001) were all significant in terms of effects on PFS. However, multivariate analysis revealed only advanced FIGO stage (HR 3.337, 95%CI 2.049–5.436; *P*<0.001) and non-early onset CIN (HR 5.098, 95%CI 3.389–7.669; *P*<0.001) as independent prognostic factors associated with poor PFS. Moreover, analysis for OS showed that age ≥ 50 years, advanced FIGO stage, high histological grade, lymph node involvement, sub-optimal tumor residual, CA125 level >35 U/mL, non-early onset of CIN were risk factors for OS in univariate analysis, but only advanced FIGO stage (HR 5.004, 95%CI 2.951–8.485; *P*<0.001), sub-optimal tumor residual (HR 3.182, 95%CI 1.970–5.140; *P*<0.001) and timing of CIN (HR 6.713, 95%CI 4.295–10.492; *P*<0.001) were independent prognosis factors for OS in multivariate analysis (Table 5). However, there was no correlation between severity of CIN and PFS or OS.

**Table 4:** Univariate and multivariate analysis for the association between clinical characteristics and progression-free survival

Variable	Univariate		Multivariate	
	HR(95% CI)	*P* value	HR(95% CI)	*P* value
Age (year)				
<50	1			
≥ 50	1.352(0.980–1.866)	0.066		
FIGO stage				
I-II	1		1	
III-IV	2.577(1.717–3.868)	<0.001	3.337(2.049–5.436)	<0.001
Histological grade				
Low	1			
High	1.250(0.852–1.835)	0.254		
Lymph node invasion				
Negative	1		1	
Positive	1.608(1.107–2.336)	0.013	1.069(0.692–1.653)	0.763
Tumor residual (cm)				
Optimal(≤1)	1		1	
Sub-optimal(>1)	1.732(1.204–2.466)	0.003	1.314(0.864–1.997)	0.202
CA125 level (U/mL)				
≤ 35	1		1	
>35	3.156(1.468–6.785)	0.003	1.454(0.621–3.407)	0.388
Severity of CIN				
Mild versus Absence	0.241(0.139–0.419)	<0.001	0.593(0.322–1.092)	0.093
Sever versus Absence	0.275(0.150–0.506)	<0.001	0.615(0.318–1.888)	0.148
Mild versus Sever	0.876(0.598–1.282)	0.495	0.965(0.649–1.435)	0.860
Timing of CIN				
Early onset	1		1	
Non-early onset	3.803(2.687–5.383)	<0.001	5.098(3.389–7.669)	<0.001

**Table 5:** Univariate and multivariate analysis for the association between clinical characteristics and overall survival

Variable	Univariate		Multivariate	
	HR(95% CI)	*P* value	HR(95% CI)	*P* value
Age (year)				
<50	1		1	
≥ 50	1.598(1.147–2.226)	0.006	1.264(0.887–1.802)	0.195
FIGO stage				
I-II	1		1	
III-IV	3.794(2.454–5.864)	<0.001	5.004(2.951–8.485)	<0.001
Histological grade				
Low	1		1.	
High	1.613(1.073–2.425)	0.022	1.302(0.823–2.059)	0.259
Lymph node invasion				
Negative	1		1	
Positive	2.583(1.759–3.795)	<0.001	1.042(0.642–1.694)	0.867
Tumor residual (cm)				
Optimal(≤1)	1		1	
Sub-optimal(>1)	4.183(2.845–6.149)	<0.001	3.182(1.970–5.140)	<0.001
CA125 level (U/mL)				
≤ 35	1		1	
>35	4.360(1.779–10.681)	0.001	1.142(0.411–3.168)	0.799
Severity of CIN				
Mild versus Absence	0.224(0.128–0.393)	<0.001	0.512(0.269–0.975)	0.042
Sever versus Absence	0.211(0.112–0.397)	<0.001	0.493(0.246–0.989)	0.046
Mild versus Sever	1.060(0.708–1.588)	0.776	1.039(0.671–1.608)	0.864
Timing of CIN				
Early onset	1		1	
Non-early onset	3.696(2.593–5.268)	<0.001	6.713(4.295–10.492)	<0.001

## Discussion

Patients who undergo carboplatin plus paclitaxel adjuvant chemotherapy experience different levels and types of adverse effects. Neutropenia is the most evident adverse effects of chemotherapy. Since 2013, several investigations represented that timing of CIN may predict chemotherapeutic response or survival [12–16]. The present investigation, to our best knowledge, is the first report on the association between timing of CIN and chemotherapeutic response or survival in SOC patients. A significantly better chemotherapeutic response and survival outcomes were observed in patients who had early onset CIN as compared to that of non-early onset. Consistent with previous researches, our study provides evidences that the timing of CIN onset can be exploited for prediction of chemotherapeutic response and survival. For example, the chemoresistance incident was more likely to occur in non-early onset neutropenia (78.33% vs. 9.17%; *P*<0.001). In addition, early onset of CIN leads to significantly improved PFS as well as OS than the non-early onset group. The median PFS in early onset neutropenia group was 23 months as compared to 9 months in case of non-early onset group(*P*<0.001), and similarly the median OS were 55 and 24 months, in the respective groups (*P*<0.001).

Several studies with different types of cancer have demonstrated the effect of CIN on the improved survival of patients. Rocconi et al. [19] first reported the association of CIN and survival in 255 primary EOC patients treated with 6 cycles of platinum plus taxane regimen. However, Kim et al. [11] reported that CIN as a non-significant prognostic indicator in ovarian cancer patients treated with carboplatin plus paclitaxel. In 2013, Jang SH et al. [12] have provided the viewpoint that the timing of CIN onset following chemotherapy can be a determinant of survival against metastatic non-small cell lung cancer. Similar relations were also found in pancreatic [14], gastric [15], and metastatic colon cancer [16]. This study demonstrates that early onset CIN is a predictor of better survival outcomes against SOC. This may be a due to chemotherapy induced effective killing of residual as well as cancer stem cells. It suggested that CIN reflects the pharmacokinetics of cytotoxic drugs, the genetic predisposition of the patients, and inflammation in the tumor microenvironment, which are the common factors related to survival outcomes.

First, CIN reflects the dose and pharmacokinetics of chemotherapy regimen. In practice, the cytotoxic drugs dosing is based on body-surface area (BSA). Several reports have showed that this method of selecting dose may be insufficient or suboptimal in some patients due to the uncertain correlation between the pharmacokinetics of many cytotoxic drugs and BSA [20]. Differences in metabolisms, drug distribution, and catabolism affects the plasma concentration of cytotoxic drugs which may lead to variation in therapeutic effectiveness due to under-dosing with standard chemotherapy [21]. However, it is evident that at least the cornerstone of the medical treatment of ovarian cancer patients, the carboplatin, is not dosed based on the BSA but on AUC. Therefore, lack of prognostic value of CIN in ovarian cancer might be explained by the fact that AUC dosing of carboplatin, the cornerstone of chemotherapy in this disease, prevents underdosing more than dosing strategies based on BSA [22]. Moreover, it is too expensive and not practical to assess drug plasma concentration in each patient. Therefore, based on our findings, the early onset of CIN may be a biomarker of pharmacokinetic changes, and can be used by physicians for adjustment of drug dose.

Second, patient’s genetic predisposition may determine tumor chemosensitivity. Theoretically all cells of a patient (including healthy cells, particularly hemopoietic cells) have similar pharmacokinetics characteristics [23]. In other words, we believe that the sensitivity to the chemotherapeutic drug in tumor cells is similar to the neutrophils in an individual patient. Our research showed that the chemoresistance incident was more likely to occur in non-early onset neutropenia, suggesting that patients with early onset CIN are chemosensitive to carboplatin and paclitaxel. On the other hand, the efficiency of cancer chemotherapy is determined by intrinsic and acquired chemoresistance [24]. The patients who do not develop neutropenia within 6 cycles in our research might be resistant to carboplatin plus paclitaxel regimen intrinsically.

Furthermore, inflammation at the tumor site are crucial for regulation of tumor development and progression [25–27]. Elevated blood neutrophil could suppress the anti-tumor immune response and promote tumor angiogenesis, resulting in speeding up tumor proliferation. Therefore, early onset CIN may slow tumor progression by releasing immune suppression and disrupting angiogenesis, resulting in better survival.

Based on the above three possible mechanisms, it is evident that early onset CIN may be a factor for predicting chemosensitivity and favorable prognosis. However, there are several limitations concerning the present research. Firstly, it was retrospective in design, with a limited sample size. Secondly, the patients enrolled in the study belong to same ethnicity and received a single chemotherapy regimen, carboplatin plus paclitaxel. Despite these drawbacks, the study forms the lead for an accurate and easily measurable surrogate marker for predicting chemotherapeutic response and prognosis of ovarian cancer.

## Conclusion

The findings of the research suggest that early onset CIN may be used to predict chemosensitivity and favorable prognosis in SOC patients receiving carboplatin plus paclitaxel adjuvant chemotherapy post cytoreductive surgery. However, a large-scale multicentric study would be essential to fully elucidate the association of timing of CIN onset and effective chemotherapy.

## Data Availability

The dataset used and analysed during the present study is available from the corresponding author upon reasonable request.

## Abbreviations

ANC: Absolute neutrophil count
BSA: Body-surface area
CI: Confidence interval
CIN: Chemotherapy-induced neutropenia
EOC: Epithelial ovarian cancer
HR: Hazard ratio
OR: Odds ratio
OS: Overall survival
PFS: Progression free survival
SOC: Serous ovarian cancer
